# A Phase 1, Randomized, Open‐Label, Parallel Group Study to Evaluate the Relative Bioavailability and Safety of Subcutaneous Bepirovirsen when Delivered from a Vial or Prefilled Syringe Fitted with a Safety Syringe Device in Healthy Adult Participants

**DOI:** 10.1002/cpdd.1615

**Published:** 2025-10-14

**Authors:** Amir S. Youssef, Poonam Shah, Maxwell Hu, Helene Plein, Abhishek Roy, Ravi Sharma, Sarah Mole, Magdalena Blazejczyk, Wendy Cross, Brian Spears, Samuel Pak, Rejbinder Kaur, Robert Elston, Dickens Theodore, Marjan Hezareh, Ahmed Nader

**Affiliations:** ^1^ Clinical Pharmacology and Quantitative Medicine GSK Collegeville Pennsylvania USA; ^2^ Clinical Development GSK Stevenage UK; ^3^ Research and Development GSK Shanghai China; ^4^ Safety Evaluation and Risk Management GSK London UK; ^5^ Development Biostatistics GSK Bangalore India; ^6^ Development Biostatistics GSK Warsaw Poland; ^7^ Safety Evaluation and Risk Management GSK Warsaw Poland; ^8^ Medicine Development Industrialisation GSK Stevenage UK; ^9^ Clinical Research ‐ Early Clinical Development PPD Austin Texas USA; ^10^ Clinical Research ‐ Early Clinical Development PPD Las Vegas Nevada USA; ^11^ Development Global Clinical Operations GSK Stevenage UK; ^12^ Clinical Development GSK Durham North Carolina USA; ^13^ Clinical Development GSK London UK

**Keywords:** antisense oligonucleotide, chronic hepatitis B, HBV DNA, pharmacokinetics, self‐administration

## Abstract

Bepirovirsen, an antisense oligonucleotide in development for the treatment of chronic hepatitis B virus (HBV) infection, is administered from glass vials as a subcutaneous (SC) injection by healthcare professionals (HCPs). A ready‐to‐use prefilled syringe (PFS) assembled with a safety syringe device (SSD) has been developed to make administration more convenient and facilitate patient self‐administration. This Phase 1, open‐label, randomized, parallel‐group study evaluated the relative bioavailability of bepirovirsen delivered from a vial or PFS SSD, assessed the viability of PFS SSD self‐administration, and evaluated the safety and tolerability of SC bepirovirsen in healthy participants. Participants (N = 159) received a single 300 mg SC dose of bepirovirsen administered by a HCP (vial [n = 46] or PFS SSD [n = 49]), or self‐administered (PFS SSD, with [n = 32] or without [n = 32] training from a HCP). Relative bioavailability (primary endpoint) of HCP‐administered bepirovirsen delivered by vial versus PFS SSD was assessed using maximum observed plasma concentration (C_max_) and area under the concentration–time curve from time zero extrapolated to infinity (AUC_(0‐inf)_). Participants were monitored for adverse events. Bepirovirsen exposure was bioequivalent when HCP‐administered either by vial or PFS SSD; the 90% confidence intervals (CIs) for the geometric mean ratios (GMRs) were within the standard bioequivalence reference range, 0.80‐1.25, for both C_max_ (1.02 [0.91‐1.14]) and AUC_(0‐inf)_ (1.05 [0.96‐1.15]). Self‐administration using PFS SSD achieved bioequivalence for bepirovirsen exposure compared with HCP administration. No new safety concerns were identified. These findings confirm that PFS SSD is a viable alternative to vials for bepirovirsen administration, when HCP‐ or self‐administered, for the treatment of chronic HBV.

**Clinical trial identifier**: NCT06058390

Approximately 254 million people were affected by chronic hepatitis B virus (HBV) infection globally in 2022, and an estimated 1.1 million died from the disease.^1^, ^2^, ^3^ Bepirovirsen (GSK3228836) is an antisense oligonucleotide (ASO) currently being evaluated in the B‐Well Phase 3 clinical trial program (NCT05630807; NCT05630820) for the treatment of chronic HBV infection.^4^, ^5^, ^6^, ^7^ Bepirovirsen is designed to target and bind to a conserved 20‐nucleotide region present in all HBV messenger RNA (mRNA) molecules, thereby inducing RNA degradation and a reduction in the levels of viral DNA and viral proteins such as hepatitis B surface antigen (HBsAg).^4^ In Phase 2b trials, bepirovirsen has been shown to induce durable HBsAg and HBV DNA loss after the end of treatment, when administered either alone or on a background of nucleos(t)ide analogs (NA), or when administered sequentially with pegylated interferon on a background of NA.^5^, ^8^ The bepirovirsen long‐term follow‐up study, B‐Sure (NCT04954859; ongoing), showed up to 2 years’ durability of functional cure (defined as sustained HBsAg loss and HBV DNA below the lower limit of quantification at 24 weeks off treatment^9^) after the end of bepirovirsen treatment.^10^, ^11^

In clinical trials, bepirovirsen is administered once weekly for up to 24 weeks as a subcutaneous (SC) injection from a clear glass vial by a healthcare professional (HCP).^4^, ^5^ However, studies suggest that self‐administration of injections is more convenient for patients, increases treatment adherence, and can benefit patients through reduced hospital visits and associated costs.^12^, ^13^, ^14^ In light of this, a ready‐to‐use prefilled syringe (PFS) assembled with a safety syringe device (SSD) has been developed to make administration more convenient, facilitate patient self‐administration, and further reduce the risk of needle stick injuries that is still associated with prefilled syringes without integrated safety features.^15^ The formulation of bepirovirsen is identical between the vial and the PFS SSD.

The current study was designed to evaluate the relative bioavailability of bepirovirsen when delivered via SC injection from a vial (HCP‐administered) or by a PFS SSD (HCP‐ or self‐administered) in healthy adult participants. Safety and tolerability were assessed as a secondary objective.

## Methods

### Study design

The study was conducted in accordance with the Declaration of Helsinki, the Council for International Organizations of Medical Sciences, International Ethical Guidelines, the Good Clinical Practice guidelines of the International Council for Harmonization, and other applicable laws and regulations. An investigational review board (IRB) committee approved the study protocol (Advarra IRB, 6100 Merriweather Drive, Suite 600, Columbia, Maryland, 21044, United states). Written informed consent was obtained from all participants prior to study enrollment.

**Figure 1:**
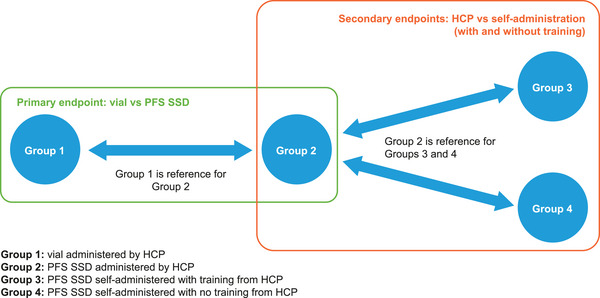
Study design. ATP, according to protocol; HCP, healthcare professional; ITT, intent to treat; PFS SSD, prefilled syringe assembled with a safety syringe device.

This was a Phase 1, open‐label, randomized, parallel‐group study (219239, NCT06058390). Participants were randomized 3:3:2:2 to receive a single dose of bepirovirsen 300 mg either administered by a HCP from a vial (Group 1), administered by a HCP using the PFS SSD (Group 2), self‐administered using the PFS SSD with training from a HCP (Group 3), or self‐administered using the PFS SSD without training from a HCP (Group 4) (Figure 1). Participants in Groups 3 and 4 were provided with instructions for use and monitored by a HCP during self‐administration. The randomization was stratified by body weight (50‐<70 kg, 70‐<80 kg, and ≥80 kg). The site of injection was randomized in a 1:1:1 ratio to the upper arm, abdomen, or thigh in Groups 1 and 2, and in a 1:1 ratio to abdomen or thigh in Groups 3 and 4. Pharmacokinetic (PK) samples were collected from all participants pre‐dose and at 1, 1.5, 2, 3, 4, 5, 6, 8, 12, 24, 48, 72, 168, 336, 672, 1008, and 1512 h post‐dose. PK samples were assayed for bepirovirsen in plasma using a validated liquid‐liquid extraction assay following high performance liquid chromatography with MS/MS detection. All participants were monitored for adverse events (AEs) throughout.

### Participants

Healthy male and female participants, aged between 18 and 55 years, were enrolled from 2 centers in the United States. Participants were required to have body weight ≥50 kg and body mass index (BMI) within 19‐29.9 kg/m^2^. Participants were excluded if they had: a history or presence of cardiovascular, respiratory, hepatic, renal, gastrointestinal, endocrine, hematologic, or neurological disorders capable of significantly altering the absorption, metabolism, or elimination of drugs; or any malignancy within the past 5 years; platelets <140×10^9^/L; alanine aminotransferase (ALT) >1.5× upper limit of normal (ULN), or total bilirubin >1.5×ULN; a current or chronic history of liver disease or known hepatic or biliary abnormalities; or prior treatment with any oligonucleotide or siRNA within 12 months before dosing.

### Treatments

All participants received a single dose of bepirovirsen 300 mg either by vial or PFS SSD, given as two 150 mg SC injections that were given as close together in time as possible (within 5 min) and spaced at least 5 cm apart.

The bepirovirsen PFS SSD consisted of a sterile liquid drug product in a single‐use clear glass PFS assembled into an SSD that delivered 1 mL of 150 mg/mL bepirovirsen for SC administration. The SSD was a single‐use, disposable device designed to enable manual delivery by a patient, caregiver, or HCP, while providing post‐use passive sharps injury protection via a needle shielding feature, and enhanced ergonomics. The needle shielding mechanism was governed by a spring which automatically shielded the needle once the contents of the syringe were dispensed and pressure was released from the plunger rod.

### Endpoints and assessments

The primary endpoint was to estimate the relative bioavailability of a single dose of bepirovirsen delivered by SC injection by a HCP in healthy participants using a vial versus PFS SSD. This was assessed using bepirovirsen maximum observed plasma concentration (C_max_) and area under the concentration–time curve from time zero extrapolated to infinity (AUC(_0‐inf_)), which were estimated by non‐compartmental PK methods based on the individual concentration–actual time data using Phoenix WinNonlin version 8.1.1, using Group 1 as reference for Group 2. The secondary endpoint was to estimate the relative bioavailability of a single dose of bepirovirsen delivered by SC injection using the PFS SSD when HCP‐administered versus self‐administered with/without HCP training in healthy participants. This was measured using C_max_ and AUC_(0‐inf)_ of bepirovirsen in plasma (Group 2 as reference for Groups 3 and 4). Safety and tolerability of single doses of bepirovirsen 300 mg by randomized group were assessed using occurrence of AEs, serious AEs, AEs of special interest (AESIs; these included injection site reactions, ALT increase, thrombocytopenia, renal injury, vascular inflammation, and complement activation), and change from baseline in laboratory tests and vital signs. MedDRA preferred terms captured as AESIs are listed in Table S1. Hematological monitoring was in place due to the potential 2′‐O‐methoxyethyl (2′MOE) modified ASO class effect on platelet numbers.^16^

### Sample size and statistical analysis

The planned sample size was 160 participants. Statistical analysis was performed on the natural logarithms of AUC_(0‐inf)_ and C_max_. For each of the primary and secondary endpoints, data were analyzed using separate linear fixed effects models with randomized group (i.e. Group 1 versus Group 2 for the primary analysis; Group 2 versus Group 3, and Group 2 versus Group 4 for the secondary analyses) and injection site (arm, abdomen, or thigh for Groups 1 and 2, and abdomen or thigh for Groups 3 and 4) included as categorical covariates, and natural logarithm transformed baseline weight as a continuous covariate. The form for the models is specified in the appendix. The regression parameters were estimated using least squares with a model‐based estimator of the variance. Effects were estimated and 2‐sided 90% confidence intervals (CIs) were constructed on the log scale derived from the model for the treatment differences. The point estimates and the associated 90% CIs were exponentiated to obtain estimates for geometric mean ratios (GMRs) and 90% CIs on the original scale. Bepirovirsen exposure was considered to be bioequivalent if the 90% CIs for the GMRs of both C_max_ and AUC_(0‐inf)_ fell within the standard bioequivalence reference range of 0.80 to 1.25. Study estimands are listed in Table S2.

## Results

### Patient population

**Figure 2:**
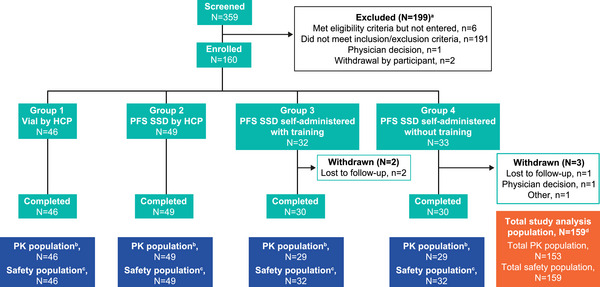
Participant disposition. HCP, healthcare professional; PFS SSD, prefilled syringe assembled with a safety syringe device; PK, pharmacokinetic. ^a^Participants may have more than one reason for exclusion; ^b^Participants were included in the PK population if they were in the safety population and received all of both the injections and had at least 1 non‐missing PK assessment. ^c^Participants were included in the safety population if they took at least 1 dose of study intervention. ^d^One participant in Group 4 did not remove the cap during dosing and did not receive any study intervention (reason for withdrawal listed as “other”); this patient was not included in any study analyses.

A total of 359 participants were screened and 160 participants enrolled in the study. In total, 5 participants across Groups 3 and 4 withdrew from the study, of whom 1 participant (Group 4) was withdrawn due to physician decision (based on failure to cooperate and complete outpatient visits), 3 participants (2 in Group 3 and 1 in Group 4) were lost to follow‐up, and 1 participant (Group 4) did not remove the cap from the PFS SSD during dosing and did not receive any study intervention. The latter participant was not included in the analysis, leading to a total study analysis population of 159 participants (Group 1, n = 46; Group 2, n = 49; Group 3, n = 32; Group 4, n = 32). Participant study status and reasons for withdrawal are shown in Figure 2. Participant demographics are summarized in Table 1. In the overall study population, 52% of participants were female, the mean (SD) age was 36.5 (9.35) years, and the mean (SD) BMI was 25.68 (2.746) kg/m^2^. The majority of participants were of White (48%) or Black/African American (35%) race and were not of Hispanic/Latino ethnicity (67%). Most of the participants were in the 50 to <70 kg (39%) and 70 to <80 kg (36%) weight categories, with similar distribution between weight categories across all 4 treatment groups.

**Table 1:** Participant demographics.

	Group 1 Vial by HCP N = 46	Group 2 PFS SSD by HCP N = 49	Group 3 PFS SSD self‐administered with training N = 32	Group 4 PFS SSD self‐administered without training N = 32	Total N = 159
**Sex**					
Female	26 (57%)	25 (51%)	15 (47%)	16 (50%)	82 (52%)
Male	20 (43%)	24 (49%)	17 (53%)	16 (50%)	77 (48%)
**Age (years)**					
Mean (SD)	35.7 (9.09)	36.5 (9.57)	36.6 (9.35)	37.5 (9.74)	36.5 (9.35)
**Ethnicity**					
Hispanic/Latino	13 (28%)	15 (31%)	10 (31%)	15 (47%)	53 (33%)
Not Hispanic/Latino	33 (72%)	34 (69%)	22 (69%)	17 (53%)	106 (67%)
**Race**					
American Indian/Alaska native	1 (2%)	1 (2%)	1 (3%)	0	3 (2%)
Asian	2 (4%)	7 (14%)	3 (9%)	1 (3%)	13 (8%)
Black/African American	14 (30%)	18 (37%)	13 (41%)	10 (31%)	55 (35%)
Multiple	3 (7%)	3 (6%)	1 (3%)	2 (6%)	9 (6%)
Native Hawaiian/Other Pacific Islander	1 (2%)	0	1 (3%)	1 (3%)	3 (2%)
White	25 (54%)	20 (41%)	13 (41%)	18 (56%)	76 (48%)
American Indian/Alaska native	1 (2%)	1 (2%)	1 (3%)	0	3 (2%)
Asian	2 (4%)	7 (14%)	3 (9%)	1 (3%)	13 (8%)
**Weight category**					
50 to <70 kg	18 (39%)	19 (39%)	12 (38%)	13 (41%)	62 (39%)
70 to <80 kg	17 (37%)	18 (37%)	12 (38%)	11 (34%)	58 (36%)
≥80 kg	11 (24%)	12 (24%)	8 (25%)	8 (25%)	39 (25%)
**BMI (kg/m^2^)**					
Mean (SD)	25.85 (2.853)	25.62 (2.744)	25.18 (3.034)	26.01 (2.307)	25.68 (2.746)

BMI, body mass index; HCP, healthcare professional; PFS SSD, prefilled syringe assembled with a safety syringe device; SD standard deviation.

### Pharmacokinetics

A total of 153 of the 159 study analysis participants were included in the PK summaries and statistical comparisons of the PK data for the primary and secondary endpoint analyses. There were 6 participants (3 in Group 3 and 3 in Group 4) who each received an incomplete dose (of either the first or second dose or both) of study intervention due to incorrect use of the PFS and were excluded from the PK analyses (Table S3).

**Figure 3:**
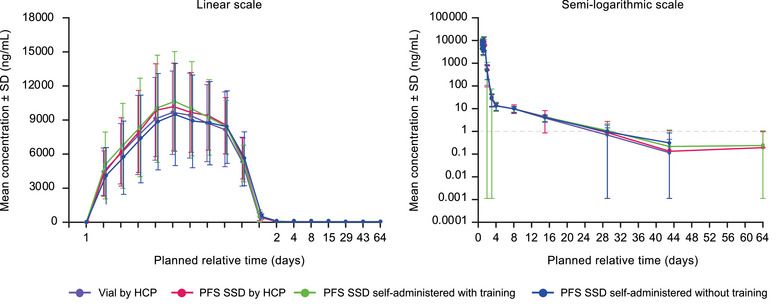
Mean bepirovirsen plasma concentration–time profiles. HCP, healthcare professional; PFS SSD, prefilled syringe assembled with a safety syringe device; SD, standard deviation; SSD, safety syringe device.

The mean bepirovirsen concentration–time profiles and the geometric mean bepirovirsen C_max_ and AUC_(0‐inf)_ values were similar across treatment arms (Figure 3 and Table 2). Analysis of the primary endpoint demonstrated the bioequivalence of bepirovirsen exposure from a vial versus PFS SSD when HCP‐administered, as the GMR (90% CI) was 1.02 (0.91, 1.14) for C_max_ and 1.05 (0.96, 1.15) for AUC_(0‐inf)_ (Table 3).

**Table 2:** Summary of bepirovirsen plasma pharmacokinetic parameters by treatment group.

	Geometric mean (% CV)			
PK parameter	Group 1 Vial HCP (N = 46)	Group 2 PFS SSD HCP (N = 49)	Group 3 PFS SSD self‐administered with training (N = 29)	Group 4 PFS SSD self‐administered without training (N = 29)
**C_max_ (ng/mL)**	9873.38 (40.96)	10,242.48 (39.54)	10,391.83 (42.70)	9516.35 (47.41)
**AUC_(0‐inf)_ (ng·h/mL)**	111,843.70 (32.93)	119,271.61 (29.70)	119,080.46 (26.28)	112,338.58 (35.01)

AUC_(0‐inf)_, area under the concentration–time curve from time zero extrapolated to infinity; C_max_, maximum observed plasma concentration; CV, coefficient of variation; HCP, healthcare professional; PFS SSD, prefilled syringe assembled with a safety syringe device; PK, pharmacokinetic.

**Table 3:** Summary of primary and secondary pharmacokinetic outcomes.

	Geometric mean		
	Reference	Test	GMR (90% CI) Test/Reference
**Primary endpoint: vial versus PFS SSD, HCP‐administration**			
**PK parameter**	**Group 1** **Vial HCP** **(N = 46)**	**Group 2** **PFS SSD HCP** **(N = 49)**	
**C_max_ (ng/mL)**	9954.89	10,163.74	1.02 (0.91, 1.14)
**AUC_(0‐inf)_ (ng·h/mL)**	112,495.66	118,622.59	1.05 (0.96, 1.15)
**Secondary endpoints: PFS SSD, HCP‐ versus self‐administration**			
	**Group 2** **PFS SSD HCP (N = 49)**	**Group 3** **PFS SSD self‐administered with training (N = 29)**	
**C_max_ (ng/mL)**	10,225.39	10,421.21	1.02 (0.88, 1.18)
**AUC_(0‐inf)_ (ng·h/mL)**	120,034.02	117,805.29	0.98 (0.88, 1.09)
	**Group 2** **PFS SSD HCP (N = 49)**	**Group 4** **PFS SSD self‐administered without training (N = 29)**	
**C_max_ (ng/mL)**	10,007.69	9896.63	0.99 (0.87, 1.13)
**AUC_(0‐inf)_ (ng·h/mL)**	117,938.68	114,492.19	0.97 (0.87, 1.08)

Estimates for geometric means and 90% CIs were obtained by exponentiating the point estimates and associated 90% CIs from the fixed effects models.

AUC_(0‐inf)_, area under the concentration–time curve from time zero extrapolated to infinity; C_max_, maximum observed plasma concentration; CI, confidence interval; GMR, geometric mean ratio; HCP, healthcare professional; PFS SSD, prefilled syringe assembled with a safety syringe device; PK, pharmacokinetic.

Analysis of the secondary PK endpoint demonstrated bioequivalence of bepirovirsen exposure following PFS SSD by HCP versus self‐administration. The GMR (90% CI) with participant training (Group 2 versus Group 3) was 1.02 (0.88, 1.18) for C_max_ and 0.98 (0.88, 1.09) for AUC_(0‐inf)_, and without participant training (Group 2 versus Group 4) was 0.99 (0.87, 1.13) for C_max_ and 0.97 (0.87, 1.08) for AUC_(0‐inf)_.

The geometric mean bepirovirsen C_max_ and AUC_(0‐inf)_ decreased with increasing body weight. Between the 50‐<70kg and ≥80kg groups, there was a decrease of 24%‐47% in geometric mean C_max_ and a decrease of 19%‐43% in geometric mean AUC_(0‐inf)_.

Some differences were seen between different injection sites (Table 4). In Groups 1 and 2 the geometric mean C_max_ tended to be lower when administered in the thigh compared with the abdomen and upper arm. Across all treatment groups the geometric mean C_max_ of bepirovirsen tended to be lower when administered in the thigh compared with the other injection sites.

**Table 4:** Summary of bepirovirsen plasma pharmacokinetic parameters by treatment arm and injection site.

		Group 1 Vial HCP (N = 46)		Group 2 PFS SSD HCP (N = 49)		Group 3 PFS SSD self‐administered with training (N = 29)		Group 4 PFS SSD self‐administered without training (N = 29)	
**PK parameter**	**Injection site**	N	Geometric mean (95% CI)	N	Geometric mean (95% CI)	N	Geometric mean (95% CI)	N	Geometric mean (95% CI)
**C_max_ (ng/mL)**	Upper arm	17	10,632.78 (8972.78, 12,599.89)	16	10,673.66 (9151.45, 12,449.06)	NA			
	Thigh	15	9020.58 (7191.85, 11,314.33)	16	8958.78 (7599.47, 10,561.22)	15	8364.11 (6803.96, 10,282.00)	15	7418.52 (5871.13, 9373.74)
	Abdomen	14	9940.82 (7649.50, 12,918.48)	17	11175.99 (8685.39, 14,380.79)	14	13,112.89 (10,940.04, 15,717.28)	14	12,426.49 (10,387.73, 14,865.39)
**AUC_(0‐inf)_ (ng·h/mL)**	Upper arm	17	121,016.40 (105,264.27, 139,125.73)	16	119,168.08 (105,722.32, 134,323.88)	NA			
	Thigh	15	109,819.96 (90,552.56, 133,187.00)	16	115,836.75 (104,042.66, 128,967.79)	15	109,286.11 (97,224.08, 122,844.60)	15	93,750.29 (79,778.96, 110,168.35)
	Abdomen	14	103,642.99 (84,827.14, 126,632.46)	17	122,697.65 (99,474.72, 151,342.11)	14	130,550.51 (111,090.73, 153,419.06)	14	136,362.97 (115,885.28, 160,459.20)

AUC_(0‐inf)_, area under the concentration–time curve from time zero extrapolated to infinity; C_max_, maximum observed plasma concentration; CI, confidence interval; HCP, healthcare professional; NA, not applicable; PFS SSD, prefilled syringe assembled with a safety syringe device; PK, pharmacokinetic.

### Safety

A total of 94% (150/159) of participants reported AEs, with similar proportions of participants reporting AEs across all treatment groups (88‐100%; Table 5 and Table S4). Most AEs were considered related to study drug by the investigator (148/159 participants [93%]; Table S5). There were no serious or fatal AEs. Most AEs were Grade 1/2 in severity; <10% of participants reported Grade 3 AEs, and no Grade 4, Grade 5, or unknown Grade AEs were reported.

**Table 5:** Summary of adverse events by preferred term reported in at least 3% of participants overall (safety population).

Preferred term	Group 1 Vial by HCP N = 46	Group 2 PFS SSD by HCP N = 49	Group 3 PFS SSD self‐administered with training N = 32	Group 4 PFS SSD self‐administered without training N = 32	Total N = 159
**Any event**	**45 (98%)**	**43 (88%)**	**32 (100%)**	**30 (94%)**	**150 (94%)**
Injection site erythema	35 (76%)	37 (76%)	30 (94%)	30 (94%)	132 (83%)
Injection site swelling	21 (46%)	24 (49%)	14 (44%)	19 (59%)	78 (49%)
Injection site pain	23 (50%)	22 (45%)	10 (31%)	12 (38%)	67 (42%)
Injection site pruritus	8 (17%)	11 (22%)	9 (28%)	13 (41%)	41 (26%)
Injection site discoloration	7 (15%)	5 (10%)	6 (19%)	5 (16%)	23 (14%)
Injection site pallor	6 (13%)	3 (6%)	5 (16%)	3 (9%)	17 (11%)
Injection site hemorrhage	5 (11%)	2 (4%)	3 (9%)	1 (3%)	11 (7%)
Injection site bruising	2 (4%)	3 (6%)	4 (13%)	1 (3%)	10 (6%)
Injection site warmth	3 (7%)	2 (4%)	2 (6%)	0	7 (4%)
Pyrexia	3 (7%)	2 (4%)	1 (3%)	1 (3%)	7 (4%)
Chills	2 (4%)	0	1 (3%)	2 (6%)	5 (3%)
Headache	12 (26%)	5 (10%)	5 (16%)	7 (22%)	29 (18%)
Dizziness	1 (2%)	2 (4%)	1 (3%)	1 (3%)	5 (3%)
Nausea	3 (7%)	3 (6%)	2 (6%)	1 (3%)	9 (6%)
Nasopharyngitis	2 (4%)	1 (2%)	1 (3%)	0	4 (3%)
Myalgia	5 (11%)	1 (2%)	1 (3%)	1 (3%)	8 (5%)
Contusion	1 (2%)	2 (4%)	0	1 (3%)	4 (3%)

HCP, healthcare professional; PFS SSD, prefilled syringe assembled with a safety syringe device.

AESIs made up the majority of AEs reported, largely due to injection site reactions (148/159 [93%]). Most of these events were Grade 1 or 2 and had resolved by the end of the study. The proportion of participants experiencing injection site reactions was higher when PFS SSD was self‐administered (Group 3: 32/32 [100%], Group 4: 30/32 [94%]) compared with HCP administration (Group 2: 43/49 [88%]). Similar AESI profiles were observed across the 4 treatment groups. No participant was hospitalized or withdrew from the study due to any AESI. AESIs related to thrombocytopenia were reported by 18% (29/159) of participants with no clear difference between treatment groups; AESIs of thrombocytopenia reported in ≥3% of participants were injection site hemorrhage (n = 11 [7%]), injection site bruising (n = 10 [6%]) and contusion (n = 4 [3%]). AESIs related to vascular inflammation and complement activation were reported in 9% (14/159) of participants; vascular inflammation and complement activation AESIs that were reported in ≥2 participants included preferred terms of pruritus (n = 2 [1%]), medical device site dermatitis (n = 2 [1%]), and drug hypersensitivity (n = 2 [1%]). No single AE within this AESI category was reported in more than 1% (n = 2) of participants and no events met criteria for drug‐induced vascular injury. A total of 2 participants reported events within the AESI of ALT increases; 1 participant in the HCP‐administered PFS SSD group, and 1 participant in the self‐administered PFS SSD with training group each reported an AE of ALT increase and aspartate aminotransferase increase. Both ALT increases were <5× ULN. One participant in the self‐administered PFS SSD without training group reported a Grade 3 platelet decline to <100×10^9^/L. This occurred 65 days after study‐dose administration, the participant was asymptomatic and had no clinical bleeding. The event was reported as resolved. No participant reported AESI related to renal injury.

## Discussion

This study showed that bepirovirsen exposure was bioequivalent when administered by a HCP either by PFS with SSD or by vial. Additionally, self‐administration with and without prior training using the PFS SSD achieved bioequivalence for bepirovirsen exposure compared with HCP administration. Furthermore, these results show that the viscosity of the bepirovirsen formulation does not impact its bioavailability when administered by PFS SSD.

Bepirovirsen exposure decreased with increasing weight, which is consistent with previous PK population modeling^17^; however, based on pharmacokinetic–pharmacodynamic (PKPD) relationship, it is not expected to be clinically relevant. Simulations using fixed dose and different bodyweight did not result in changes in efficacy using the PKPD model. Bepirovirsen exposure differed by SC injection site, with lower exposure observed when the SC injection was administered in the thigh compared with the abdomen or upper arm. It is unclear from the data whether the difference in exposure by injection site is real or due to variability. This will be further assessed in Phase 3 studies using population PK modeling. A difference in exposure by injection site has been reported previously for other drugs.^18^ The significance of this difference in exposure on efficacy will be determined using PKPD relationship data in Phase 3.

No new safety concerns for bepirovirsen were identified. Vial and PFS SSD administration, when administered by HCPs, produced similar AE profiles. The frequency of Grade 3 AEs observed in the current study was similar to that reported in a Phase 2b bepirovirsen trial (B‐Clear; 7‐14%).^5^ Injection site reactions were the most common AE, irrespective of treatment group. Overall, injection site reactions were more common in self‐administered groups than in the group receiving PFS SSD administration by HCPs; however, injection site pain was reported by more participants in the HCP‐administered treatment groups than in the self‐administered treatment groups. These differences were not considered clinically meaningful, and are in line with a previous study where patients reported that self‐administration SC injection allows for greater convenience and accessibility, less pain during injections, increased privacy, and fewer logistical and financial challenges than clinic‐administered intramuscular injections,^13^ though it should be acknowledged that the differences in pain perception may, in part, be due to the mode of administration (SC versus intramuscular).

The option of self‐administration of bepirovirsen could reduce the clinic visit burden for patients who live in areas where commercial diagnostic laboratories and telemedicine are prevalent. Furthermore, the use of PFS with retracted needles providing patients with needlestick protection during self‐administration may reduce healthcare‐ and patient‐related burdens, and increase adherence and patient empowerment, as has been observed for SC self‐administration versus intramuscular clinic‐administration of other drugs.^13^ While 6 participants received an incomplete dose of bepirovirsen, owing to incorrect use of the PFS (4 of them injected approximately 75% to 95% of the dose), device usability was evaluated in separate formative and summative human factors studies, and the device and instructions for use were successfully validated for self‐administration (unpublished data).

A key strength was conducting this study in healthy participants as this mitigated the need for recruitment and minimized patient burden limitations (e.g. reduced risk of AEs and fewer confounding variables such as pre‐existing conditions or medications), which are associated with studies involving patients with a disease. Additionally, by including only healthy participants in this study, data can be extrapolated to other populations. However, the use of healthy participants may also be considered a limitation because this is not the intended population for bepirovirsen; this is a common approach for relative bioavailability studies, owing to the difficulty of enrolling patients in a single‐dose study. Undertaking a single‐dose study helped to provide a sensitive model that can establish comparability between injection devices with respect to differences in the extent and rate of absorption. Finally, to prevent bias and maintain balance in treatment groups, the injection site was randomized in addition to the randomization for the device. Randomization was also stratified by body weight to minimize the impact of potential confounding factors and variability, as body weight has been previously identified as a significant covariate in the population PK model for bepirovirsen exposure.^17^

## Conclusions

The results of the study confirm that the PFS SSD is a viable alternative to using vials to administer bepirovirsen. Furthermore, self‐administration of bepirovirsen using the PFS SSD may decrease burden of treatment in chronic HBV patients.

## Authors Contributions

The authors meet criteria for authorship as recommended by the International Committee of Medical Journal Editors, take responsibility for the integrity of the work as a whole, contributed to the writing and reviewing of the manuscript, and have given final approval for the version to be published. Poonam Shah, Sarah Mole, Amir S. Youssef, Maxwell Hu, Abhishek Roy, Wendy Cross, Rejbinder Kaur, Ahmed Nader, Robert Elston, and Dickens Theodore contributed to the study concept/design. Brian Spears and Samuel Pak contributed to data acquisition. Poonam Shah, Amir S. Youssef, Marjan Hu, Abhishek Roy, Ravi Sharma, Helene Plein, Magdalena Blazejczyk, and Marjan Hezareh contributed to data analysis. Poonam Shah, Amir S. Youssef, Maxwell Hu, Helene Plein, Abhshek Roy, Magdalena Blazejczyk, Wendy Cross, Ahmed Nader, Marjan Hezareh, and Dickens Theodore contributed to data interpretation. All authors reviewed and approved the final manuscript.

## Conflicts of Interest

Abhishek Roy and Ravi Sharma are employees of GSK. Poonam Shah, Amir S. Youssef, Maxwell Hu, Helene Plein, Sarah Mole, Rejbinder Kaur, Ahmed Nader, Robert Elston, Dickens Theodore, Wendy Cross and Marjan Hezareh, are employees of and hold financial equities in GSK. Brian Spears and Samuel Pak are employees of PPD. Magdalena Blazejczyk was an employee of GSK at the time of the study.

## Funding Information

This study was funded by GSK (219239; NCT06058390).

## Supporting information



Supporting information

## Data Availability

Please refer to GSK weblink to access GSK's data sharing policies and as applicable seek anonymized subject level data via the link https://www.gsk‐studyregister.com/en/.
