# Supplementary material for: A Phase 1, Randomized, Open‐Label, Parallel Group Study to Evaluate the Relative Bioavailability and Safety of Subcutaneous Bepirovirsen when Delivered from a Vial or Prefilled Syringe Fitted with a Safety Syringe Device in Healthy Adult Participants
Source: Clin Pharmacol Drug Dev. 2025 Oct 14;15(2):e1615. doi: 10.1002/cpdd.1615 (PMC12856967; doi:10.1002/cpdd.1615)
Supplement: Supplementary file 1 — Supporting information [file CPDD-15-0-s001.docx]

**A Phase 1, randomized, open-label, parallel group study to evaluate the relative bioavailability and safety of subcutaneous bepirovirsen when delivered from a vial or prefilled syringe fitted with a safety syringe device in healthy adult participants**

Amir S. Youssef, Poonam Shah, Maxwell Hu, Helene Plein, Abhishek Roy, Ravi Sharma, Sarah Mole, Magdalena Blazejczyk, Wendy Cross, Brian Spears, Samuel Pak, Rejbinder Kaur, Robert Elston, Dickens Theodore, Marjan Hezareh, Ahmed Nader

### Table S1. Bepirovirsen AESIs

| **AESI name** | **Group of terms (MedDRA SMQ, HLT or individual PTs)** |
| --- | --- |
| **AESI** | |
| ALT increase | Drug related hepatic disorders – comprehensive search (SMQ) (*Broad)*  PT: Hepatitis B |
| Vascular inflammation and complement activation | Vasculitis SMQ (Broad)  Hypersensitivity SMQ (Broad)  Immune response protein analyses NEC (HLT) |
| Renal injury | Acute renal failure SMQ (Broad)  PTs:  Blood urine  Blood urine present  Glomerulonephritis  Hematuria  Urine albumin/creatinine ratio abnormal  Urine albumin/creatinine ratio increased |
| Thrombocytopenia | Hematopoietic thrombocytopenia SMQ (broad)  Hemorrhage terms (excluding laboratory terms) SMQ – this is a sub SMQ of hemorrhages SMQ |
| Injection site reactions | PTs:  Injection site urticaria  Injection site thrombosis  Injection site extravasation  Injection site erosion  Injection site erythema  Injection site granuloma  Injection site induration  Injection site inflammation  Injection site irritation  Injection site mass  Injection site necrosis  Injection site nodule  Injection site edema  Injection site pain  Injection site swelling  Injection site ulcer  Injection site bruising  Injection site hematoma  Injection site hemorrhage  Immediate post-injection reaction  Injection related reaction  Injection site dermatitis  Injection site eczema  Injection site hypersensitivity  Injection site rash  Injection site recall reaction  Injection site vasculitis  Injection site panniculitis  Injection site phlebitis  Injection site pruritus  Injection site abscess  Injection site anesthesia  Injection site cellulitis  Injection site discoloration  Injection site discomfort  Injection site warmth |
| Significant events | |
| Immune-mediated events | Immune-mediated/autoimmune disorders (SMQs) |
| Neutropenia | PTs:  Neutropenia  Granulocytopenia  Neutrophil count decreased  Neutrophil count abnormal  Neutrophil percentage decreased  Leukopenia |

AESI, adverse event of special interest; HLT, high-level term; MedDRA; Medical Dictionary for Regulatory Activities; NEC, not elsewhere classified; PT, preferred term; SMQ, standardized MedDRA query.

### Table S2. Study estimands

| **PRIMARY ESTIMANDS**  Primary interest: To estimate the relative bioavailability, measured by C_max_ and AUC_(0–inf)_, following a single dose of bepirovirsen via PFS SSD versus a vial in healthy adult participants  The primary PK estimands are described by the following attributes:   - Population:   - Healthy male and female participants aged 18–55 years - Treatment condition:   - Single SC dose of bepirovirsen injection administered via PFS SSD versus   - Single SC dose of bepirovirsen administered via a vial - Variables:   - C_max_ and AUC_(0–inf)_ - Analysis model:   $E\left[ \ln\left( param \right) \vert X_{1}, X_{2}, X_{3} \right]=\beta_{0}+\beta_{1}X_{1}+\beta_{2}X_{2}+\beta_{3}X_{3}$   - - Where param represents the parameter being analyzed: C_max_ or AUC_(0–inf)_   - X_1_ is a classification variable indicating randomized group (Group 1 or Group 2)   - X_2_ is a classification variable indicating injection site (Arm, Abdomen, or Thigh)   - X_3_ is the natural logarithm of baseline weight - Summary measure:   - Ratio of geometric means of PFS SSD versus vials for C_max_ and AUC_(0–inf)_ - Intercurrent events:   - Use of prohibited medication. This intercurrent event is not expected to impact the PK of bepirovirsen so a treatment policy strategy will be used, and the individual PK concentrations after the occurrence of the intercurrent event will still be included in the PK analysis   - Participants who did not receive all of both the injections and had an incomplete or erroneous dosing. A principal stratum strategy policy will be used, where the stratum of interest is the group of participants who are able to receive the complete dose   A supplemental estimand will also be analyzed as per the primary estimand, except for the intercurrent event of incomplete dosing will use a treatment policy strategy |
| --- |
| **SECONDARY ESTIMANDS**  Secondary interest: to estimate the relative bioavailability, measured by C_max_ and AUC_(0–inf)_:   1. Following a single dose of bepirovirsen via PFS SSD versus self-administration of a single dose of bepirovirsen **with HCP training** 2. Following a single dose of bepirovirsen via PFS SSD versus self-administration of a single dose of bepirovirsen **with no HCP training**   The secondary PK estimands are described by the following attributes:   - Population:   - Healthy male and female participants aged 18–55 years - Treatment condition:   - Single SC dose of bepirovirsen administered via PFS SSD versus self-administration of a single dose of bepirovirsen with HCP training   - Single SC dose of bepirovirsen administered via PFS SSD versus self-administration of a single dose of bepirovirsen with no HCP training - Variables:   - C_max_ and AUC_(0–inf)_ - Analysis model:   $E\left[ \ln\left( param \right) \vert X_{1}, X_{2}, X_{3} \right]=\beta_{0}+\beta_{1}X_{1}+\beta_{2}X_{2}+\beta_{3}X_{3}$   - - Where param represents the parameter being analyzed: C_max_ or AUC_(0–inf)_   - X_1_ is a classification variable indicating randomized group (Group 2 or Group 3 for secondary analysis 1; Group 2 or Group 4 for secondary analysis 2)   - X_2_ is a classification variable indicating injection site (Arm, Abdomen, or Thigh)   - X_3_ is the natural logarithm of baseline weight - Summary measure:   - Ratio of geometric means of PFS SSD versus self-administration with HCP training and with no HCP training for C_max_ and AUC_(0–inf)_ - Intercurrent events:   - Use of prohibited medication. This intercurrent event is not expected to impact the PK of bepirovirsen so a treatment policy strategy will be used, and the individual PK concentrations after the occurrence of the intercurrent event will still be included in the PK analysis   - Participants who did not receive all of both the injections and had an incomplete or erroneous dosing. A principal stratum strategy policy will be used, where the stratum of interest is the group of participants who are able to receive the complete dose   Supplemental estimands will also be analyzed as per the secondary estimand, except for the intercurrent event of incomplete dosing will use a treatment policy strategy |

AUC_(0–inf)_, area under the concentration–time curve from time zero extrapolated to infinity; C_max_, maximum observed plasma concentration; HCP, healthcare professional; PFS SSD, prefilled syringe assembled with a safety syringe device; PK, pharmacokinetic; SC, subcutaneous.

### Table S3. Summary of protocol deviations leading to exclusion from the PK population

| **Group** | **Protocol deviations** |
| --- | --- |
| Group 3 | **First injection**: participant administered 0.9 mL of study intervention and 0.1 mL of study intervention remained in the syringe |
| Group 3 | **First injection**: participant dispensed study intervention prior to inserting the needle and did not receive any study intervention  **Second injection**: participant did not fully insert the needle and received only 0.25 mL of study intervention |
| Group 3 | **First injection**: participant administered 0.95 mL of study intervention |
| Group 4 | **First injection**: participant withdrew the needle from the skin while still pressing the plunger and did not receive any dose of study intervention |
| Group 4 | **Second injection**: participant administered 0.75 mL of study intervention |
| Group 4 | **First injection**: participant administered 0.75 mL of study intervention  **Second injection**: participant administered 0.75 mL of study intervention |

PK, pharmacokinetic.

### Table S4. Summary of adverse events by preferred term reported in any participant (safety population)

| **Preferred term** | **Group 1**  **Vial by HCP**  **N=46** | **Group 2**  **PFS SSD by HCP**  **N=49** | **Group 3**  **PFS SSD self-administered with training N=32** | **Group 4**  **PFS SSD self-administered without training N=32** | **Total**  **N=159** |
| --- | --- | --- | --- | --- | --- |
| **Any event** | **45 (98%)** | **43 (88%)** | **32 (100%)** | **30 (94%)** | **150 (94%)** |
| Injection site erythema | 35 (76%) | 37 (76%) | 30 (94%) | 30 (94%) | 132 (83%) |
| Injection site swelling | 21 (46%) | 24 (49%) | 14 (44%) | 19 (59%) | 78 (49%) |
| Injection site pain | 23 (50%) | 22 (45%) | 10 (31%) | 12 (38%) | 67 (42%) |
| Injection site pruritus | 8 (17%) | 11 (22%) | 9 (28%) | 13 (41%) | 41 (26%) |
| Injection site discoloration | 7 (15%) | 5 (10%) | 6 (19%) | 5 (16%) | 23 (14%) |
| Injection site pallor | 6 (13%) | 3 (6%) | 5 (16%) | 3 (9%) | 17 (11%) |
| Injection site hemorrhage | 5 (11%) | 2 (4%) | 3 (9%) | 1 (3%) | 11 (7%) |
| Injection site bruising | 2 (4%) | 3 (6%) | 4 (13%) | 1 (3%) | 10 (6%) |
| Injection site warmth | 3 (7%) | 2 (4%) | 2 (6%) | 0 | 7 (4%) |
| Pyrexia | 3 (7%) | 2 (4%) | 1 (3%) | 1 (3%) | 7 (4%) |
| Chills | 2 (4%) | 0 | 1 (3%) | 2 (6%) | 5 (3%) |
| Injection site inflammation | 1 (2%) | 0 | 2 (6%) | 0 | 3 (2%) |
| Injection site nodule | 2 (4%) | 0 | 0 | 1 (3%) | 3 (2%) |
| Fatigue | 1 (2%) | 0 | 1 (3%) | 0 | 2 (1%) |
| Medical device site dermatitis | 1 (2%) | 0 | 1 (3%) | 0 | 2 (1%) |
| Feeling of body temperature change | 0 | 1 (2%) | 0 | 0 | 1 (<1%) |
| Influenza like illness | 0 | 0 | 0 | 1 (3%) | 1 (<1%) |
| Injection site dermatitis | 0 | 0 | 0 | 1 (3%) | 1 (<1%) |
| Injection site discomfort | 0 | 1 (2%) | 0 | 0 | 1 (<1%) |
| Injection site exfoliation | 0 | 0 | 0 | 1 (3%) | 1 (<1%) |
| Injection site paresthesia | 0 | 0 | 0 | 1 (3%) | 1 (<1%) |
| Injection site scab | 0 | 0 | 0 | 1 (3%) | 1 (<1%) |
| Malaise | 0 | 0 | 0 | 1 (3%) | 1 (<1%) |
| Vessel puncture site reaction | 0 | 1 (2%) | 0 | 0 | 1 (<1%) |
| Headache | 12 (26%) | 5 (10%) | 5 (16%) | 7 (22%) | 29 (18%) |
| Dizziness | 1 (2%) | 2 (4%) | 1 (3%) | 1 (3%) | 5 (3%) |
| Presyncope | 2 (4%) | 1 (2%) | 0 | 0 | 3 (2%) |
| Dysgeusia | 0 | 0 | 0 | 1 (3%) | 1 (<1%) |
| Syncope | 1 (2%) | 0 | 0 | 0 | 1 (<1%) |
| Nausea | 3 (7%) | 3 (6%) | 2 (6%) | 1 (3%) | 9 (6%) |
| Diarrhea | 1 (2%) | 0 | 0 | 1 (3%) | 2 (1%) |
| Vomiting | 0 | 0 | 2 (6%) | 0 | 2 (1%) |
| Abdominal discomfort | 0 | 1 (2%) | 0 | 0 | 1 (<1%) |
| Abdominal pain | 0 | 1 (2%) | 0 | 0 | 1 (<1%) |
| Aphthous ulcer | 1 (2%) | 0 | 0 | 0 | 1 (<1%) |
| Constipation | 0 | 0 | 1 (3%) | 0 | 1 (<1%) |
| Dyspepsia | 1 (2%) | 0 | 0 | 0 | 1 (<1%) |
| Nasopharyngitis | 2 (4%) | 1 (2%) | 1 (3%) | 0 | 4 (3%) |
| Viral infection | 0 | 1 (2%) | 1 (3%) | 0 | 2 (1%) |
| Bacterial vaginosis | 1 (2%) | 0 | 0 | 0 | 1 (<1%) |
| Folliculitis | 0 | 0 | 0 | 1 (3%) | 1 (<1%) |
| Impetigo | 0 | 1 (2%) | 0 | 0 | 1 (<1%) |
| Influenza | 0 | 0 | 1 (3%) | 0 | 1 (<1%) |
| Paronychia | 1 (2%) | 0 | 0 | 0 | 1 (<1%) |
| Upper respiratory tract infection | 0 | 1 (2%) | 0 | 0 | 1 (<1%) |
| Urinary tract infection | 0 | 0 | 1 (3%) | 0 | 1 (<1%) |
| Viral upper respiratory tract infection | 0 | 0 | 1 (3%) | 0 | 1 (<1%) |
| Vulvovaginal mycotic infection | 1 (2%) | 0 | 0 | 0 | 1 (<1%) |
| Myalgia | 5 (11%) | 1 (2%) | 1 (3%) | 1 (3%) | 8 (5%) |
| Back pain | 3 (7%) | 0 | 0 | 0 | 3 (2%) |
| Groin pain | 0 | 0 | 0 | 1 (3%) | 1 (<1%) |
| Musculoskeletal discomfort | 0 | 0 | 1 (3%) | 0 | 1 (<1%) |
| Pain in extremity | 0 | 1 (2%) | 0 | 0 | 1 (<1%) |
| Contusion | 1 (2%) | 2 (4%) | 0 | 1 (3%) | 4 (3%) |
| Injection related reaction | 0 | 0 | 0 | (3%) | 1 (<1%) |
| Ligament sprain | 1 (2%) | 0 | 0 | 0 | 1 (<1%) |
| Muscle strain | 1 (2%) | 0 | 0 | 0 | 1 (<1%) |
| Skin abrasion | 1 (2%) | 0 | 0 | 0 | 1 (<1%) |
| Cough | 0 | 0 | 1 (3%) | 1 (3%) | 2 (1%) |
| Epistaxis | 1 (2%) | 1 (2%) | 0 | 0 | 2 (1%) |
| Throat irritation | 0 | 1 (2%) | 1 (3%) | 0 | 2 (1%) |
| Rhinorrhea | 0 | 0 | 0 | 1 (3%) | 1 (<1%) |
| Sneezing | 0 | 0 | 0 | 1 (3%) | 1 (<1%) |
| Throat tightness | 0 | 0 | 0 | 1 (3%) | 1 (<1%) |
| C-reactive protein increased | 2 (4%) | 0 | 1 (3%) | 0 | 3 (2%) |
| Alanine aminotransferase increased | 0 | 1 (2%) | 1 (3%) | 0 | 2 (1%) |
| Aspartate aminotransferase increased | 0 | 1 (2%) | 1 (3%) | 0 | 2 (1%) |
| Hemoglobin decreased | 0 | 0 | 0 | 1 (3%) | 1 (<1%) |
| Neutrophil percentage increased | 0 | 0 | 1 (3%) | 0 | 1 (<1%) |
| Weight increased | 0 | 0 | 1 (3%) | 0 | 1 (<1%) |
| Pruritus | 0 | 0 | 1 (3%) | 1 (3%) | 2 (1%) |
| Dermatitis atopic | 1 (2%) | 0 | 0 | 0 | 1 (<1%) |
| Dermatitis contact | 0 | 1 (2%) | 0 | 0 | 1 (<1%) |
| Drug eruption | 1 (2%) | 0 | 0 | 0 | 1 (<1%) |
| Papule | 0 | 0 | 0 | 1 (3%) | 1 (<1%) |
| Photosensitivity reaction | 1 (2%) | 0 | 0 | 0 | 1 (<1%) |
| Thrombocytopenia | 1 (2%) | 0 | 0 | 2 (6%) | 3 (2%) |
| Lymphadenopathy | 0 | 0 | 1 (3%) | 1 (3%) | 2 (1%) |
| Drug hypersensitivity | 1 (2%) | 0 | 1 (3%) | 0 | 2 (1%) |
| Allergy to arthropod sting | 1 (2%) | 0 | 0 | 0 | 1 (<1%) |
| Seasonal allergy | 0 | 0 | 1 (3%) | 0 | 1 (<1%) |
| Decreased appetite | 1 (2%) | 1 (2%) | 0 | 0 | 2 (1%) |
| Increased appetite | 0 | 0 | 2 (6%) | 0 | 2 (1%) |
| Dysuria | 0 | 1 (2%) | 0 | 0 | 1 (<1%) |
| Pollakiuria | 0 | 0 | 1 (3%) | 0 | 1 (<1%) |
| Urinary tract discomfort | 0 | 1 (2%) | 0 | 0 | 1 (<1%) |
| Dysmenorrhea | 0 | 0 | 0 | 1 (3%) | 1 (<1%) |
| Menstruation delayed | 0 | 1 (2%) | 0 | 0 | 1 (<1%) |
| Palpitations | 0 | 0 | 1 (3%) | 0 | 1 (<1%) |
| Conjunctival hyperemia | 1 (2%) | 0 | 0 | 0 | 1 (<1%) |
| Vision blurred | 1 (2%) | 0 | 0 | 0 | 1 (<1%) |
| Flushing | 0 | 0 | 1 (3%) | 0 | 1 (<1%) |

HCP, healthcare professional; PFS SSD, prefilled syringe assembled with a safety syringe device.

### Table S5. Summary of treatment-related adverse events as determined by the investigator by preferred term (safety population)

| **Preferred term** | **Group 1**  **Vial by HCP**  **N=46** | **Group 2**  **PFS SSD by HCP**  **N=49** | **Group 3**  **PFS SSD self-administered with training N=32** | **Group 4**  **PFS SSD self-administered without training N=32** | **Total**  **N=159** |
| --- | --- | --- | --- | --- | --- |
| **Any event** | **43 (93%)** | **43 (88%)** | **32 (100%)** | **30 (94%)** | **148 (93%)** |
| Injection site erythema | 35 (76%) | 37 (76%) | 30 (94%) | 30 (94%) | 132 (83%) |
| Injection site swelling | 21 (46%) | 24 (49%) | 14 (44%) | 19 (59%) | 78 (49%) |
| Injection site pain | 23 (50%) | 22 (45%) | 10 (31%) | 12 (38%) | 67 (42%) |
| Injection site pruritus | 8 (17%) | 11 (22%) | 9 (28%) | 13 (41%) | 41 (26%) |
| Injection site discoloration | 7 (15%) | 5 (10%) | 6 (19%) | 5 (16%) | 23 (14%) |
| Injection site pallor | 6 (13%) | 3 (6%) | 5 (16%) | 3 (9%) | 17 (11%) |
| Injection site warmth | 3 (7%) | 2 (4%) | 2 (6%) | 0 | 7 (4%) |
| Pyrexia | 3 (7%) | 2 (4%) | 1 (3%) | 1 (3%) | 7 (4%) |
| Chills | 2 (4%) | 0 | 1 (3%) | 2 (6%) | 5 (3%) |
| Injection site inflammation | 1 (2%) | 0 | 2 (6%) | 0 | 3 (2%) |
| Injection site nodule | 2 (4%) | 0 | 0 | 1 (3%) | 3 (2%) |
| Fatigue | 1 (2%) | 0 | 0 | 0 | 1 (<1%) |
| Feeling of body temperature change | 0 | 1 (2%) | 0 | 0 | 1 (<1%) |
| Injection site dermatitis | 0 | 0 | 0 | 1 (3%) | 1 (<1%) |
| Injection site discomfort | 0 | 1 (2%) | 0 | 0 | 1 (<1%) |
| Injection site exfoliation | 0 | 0 | 0 | 1 (3%) | 1 (<1%) |
| Injection site paresthesia | 0 | 0 | 0 | 1 (3%) | 1 (<1%) |
| Injection site scab | 0 | 0 | 0 | 1 (3%) | 1 (<1%) |
| Malaise | 0 | 0 | 0 | 1 (3%) | 1 (<1%) |
| Headache | 10 (22%) | 4 (8%) | 5 (16%) | 4 (13%) | 23 (14%) |
| Dizziness | 1 (2%) | 1 (2%) | 1 (3%) | 0 | 3 (2%) |
| Presyncope | 1 (2%) | 1 (2%) | 0 | 0 | 2 (1%) |
| Dysgeusia | 0 | 0 | 0 | 1 (3%) | 1 (<1%) |
| Nausea | 3 (7%) | 3 (6%) | 0 | 1 (3%) | 7 (4%) |
| Abdominal pain | 0 | 1 (2%) | 0 | 0 | 1 (<1%) |
| Aphthous ulcer | 1 (2%) | 0 | 0 | 0 | 1 (<1%) |
| Diarrhea | 1 (2%) | 0 | 0 | 0 | 1 (<1%) |
| Myalgia | 5 (11%) | 0 | 1 (3%) | 1 (3%) | 7 (4%) |
| C-reactive protein increased | 2 (4%) | 0 | 1 (3%) | 0 | 3 (2%) |
| Alanine aminotransferase increased | 0 | 1 (2%) | 1 (3%) | 0 | 2 (1%) |
| Aspartate aminotransferase increased | 0 | 1 (2%) | 1 (3%) | 0 | 2 (1%) |
| Neutrophil percentage increased | 0 | 0 | 1 (3%) | 0 | 1 (<1%) |
| Thrombocytopenia | 1 (2%) | 0 | 0 | 1 (3%) | 2 (1%) |
| Lymphadenopathy | 0 | 0 | 1 (3%) | 0 | 1 (<1%) |
| Drug hypersensitivity | 1 (2%) | 0 | 1 (3%) | 0 | 2 (1%) |
| Decreased appetite | 1 (2%) | 1 (2%) | 0 | 0 | 2 (1%) |
| Drug eruption | 1 (2%) | 0 | 0 | 0 | 1 (<1%) |
| Photosensitivity reaction | 1 (2%) | 0 | 0 | 0 | 1 (<1%) |
| Injection-related reaction | 0 | 0 | 0 | 1 (3%) | 1 (<1%) |
| Throat tightness | 0 | 0 | 0 | 1 (3%) | 1 (<1%) |

HCP, healthcare professional; PFS SSD, prefilled syringe assembled with a safety syringe device.
